# Effects of Three Different Kinds of Foaming Medium on the Properties of Expanded Thermal Plastic Polyurethane Prepared via Supercritical Carbon Dioxide Foaming

**DOI:** 10.3390/polym16152224

**Published:** 2024-08-05

**Authors:** Zhou Li, Yuanyuan Li, Yingru Li

**Affiliations:** 1 College of Intelligent Systens Science and Engineering, Hubei Minzu University, Enshi 445000, China; 2000048@hbmzu.edu.cn (Z.L.); 2023026@hbmzu.edu.cn (Y.L.); 2 Key Laboratory of Green Manufacturing of Super-Light Elastomer Materials of State Ethnic Affairs Commission, Hubei Minzu University, Enshi 445000, China

**Keywords:** TPU, microcellular foam, supercritical carbon dioxide, foaming medium

## Abstract

Hot air, water, and glycerol were studied as foaming mediums for the production of ETPU to evaluate their influence on the behavior of the foam and compare the optimal particles for each of the foaming temperatures selected. The results showed that the times of water foaming and glycerol foaming were shorter by about 2/3 than with hot-air foaming. The best foaming temperatures for hot-air foaming, glycerol foaming, and water foaming are 110–115 °C, 75 °C, and 90 °C, respectively. The particles of glycerol foam have a matte appearance and their gloss is not very good. However, the particles in hot-air foaming are light, and the gloss is very satisfactory. The gloss of the surface of water-foaming particles is dim. At the same time, there is a faint matte appearance. Particles made with glycerol foaming and water foaming are more even than those made with hot-air foaming. The density of foaming materials from glycerol foaming, hot-air foaming, and water foaming are raised accordingly, while the hardness of foaming materials from glycerol foaming, water foaming, and hot-air foaming are successively increased.

## 1. Introduction

The term foaming material refers to material that can be gasified inside to produce bubbles, filling it with continuous or discrete pores. The most common category applied in industry and in daily life belongs to polymeric foaming material, which comprises a polymeric matrix with a large quantity of tiny pores inside, which are generally produced through the addition of a foaming agent. Foaming agents are mainly divided into physical foaming agents, chemical foaming agents, and composite foaming agents. Physical foaming agents include CO_2_ [1,2], cyclopentane [3], etc., while chemical foaming agents include carbamide [4], azodicarbonamide (AC) [5,6], and so on. Currently, composite blowing agents include the binary composite blowing agent (multi-component composite foaming agent (CFA) [7], ternary composite blowing agent [8], and multi-composite blowing agent [9,10]. Polymeric foaming materials exhibit excellent performance, including small density [11], sound absorption [12,13], shock absorption [14], thermal insulation [15], low cost, and material economy. Currently, the main polymer materials used for polymer foams are polystyrene [16,17], polyurethane [18,19,20], polypropylene [21,22], polyethylene terephthalate [23,24], polyvinyl chloride [25,26], etc. Among them, thermoplastic polyurethane (TPU) stands out due to its superior performance and environmental protection concepts. TPU not only possesses high hardness, excellent high tensile strength, and resistance, is transparent in color, and is easy to process [27,28,29] but is also a mature environmentally friendly material [30]. At present, TPU foam material has been widely used in healthcare [31,32], engineering [33], electronic fields [34], and so on. In recent years, TPU foam material has also gradually been applied in fields such as aerospace [35], sensors [36,37], and fire-resistant materials [38].

Supercritical carbon dioxide (ScCO_2_) foaming technology is a rapidly growing foaming technology for preparing advanced and green foamed polymeric materials, which employs ScCO_2_ as the crucial blowing agent [39,40,41,42]. Compared with traditional chemical/physical blowing agents, ScCO_2_ is a nontoxic, nonflammable, chemically inert medium with a reasonably low critical point. In addition, in the supercritical state, carbon dioxide has both the mobility and diffusivity of a gas and the solvency and density of a liquid, which ensures that ScCO_2_ could swell into other kinds of polymers like TPU. When the pressure of the CO_2_ is lower than its critical point (31.1 °C, 73.8 bar) [39], the solubility decreases rapidly, so that the dissolved CO_2_ is instantly released and rapidly expands to form foam [43]. Because the ScCO_2_ foaming process does not produce any toxic gas, it has become an environmentally friendly, safe, and inexpensive method for preparing porous materials in large-scale production [44,45]. It has been widely used in thermal/acoustic insulation, electromagnetic interference (EMI) shielding, and tissue engineering [46,47,48]. Therefore, the ScCO_2_ foaming technique has already been applied to produce TPU foams in large-scale industrial production, and these TPU foams are generally called expanded TPU (ETPU) materials.

As described above, the foaming results of ETPU are mainly affected by two key steps: the dissolution of ScCO_2_ in TPU and foaming by pressure relief. Obviously, in the process of foaming by pressure relief, various process parameters, including foaming media and temperature, play crucial roles in the performance of the final foaming ETPU materials; as the evaporation of ScCO_2_ is an endothermic process, as well as a process performing work by expansion, the physical properties of foaming media that could influence heat transfer and performance should be taken into consideration, including thermal capacity, thermal conductivity, density, etc. In this paper, intermittent ScCO_2_ foaming technology is used to prepare ETPU materials. The raw TPU particles were first saturated by ScCO_2_ and then transferred into foaming media with higher temperatures to accomplish the foaming step. Three different media were employed, including air, water, and glycerol. By evaluating the physical and mechanical properties of the obtained different ETPU products foamed in different media under different temperature, the effects of hot air, water, and glycerol on foaming behavior at different temperatures were investigated. The innovation of this paper lies in not only comparing, both horizontally and vertically, the foaming performance of ETPU under different media but also in analyzing the reasons for the differences in foaming performance from macro and micro perspectives. The results could also help adjust foaming parameters to prepare ETPU materials with different physical and mechanical properties to satisfy different requirements in industry and consumer goods.

## 2. Experiment Content

### 2.1. Experimental Materials

Thermoplastic polyurethane elastomer (TPU) was purchased from Nuoyu Chemical Asia Pacific Co., Ltd. (Hongkong, China) (model: 58887, shore hardness: 88 HA, and density: 1.13 g/cm^3^). CO_2_ gas was obtained from Cixi Jinkang Gas Co., Ltd. (Cixi, China) (purity: 99.9%). Hot air was produced by an electric heating (blast) constant-temperature drying oven (see Section 2.2 for specific information). Deionized water was prepared by passing tap water through a deionization unit that was equipped with ion exchange resins, which effectively removed the cations and anions to produce high-purity water suitable for experimental use. Glycerol was bought from Sinopharm Chemical Reagent Co., Ltd. (Shanghai, China) (model: G6201; purity: 99%).

### 2.2. Experimental Equipment

**Figure 1:**
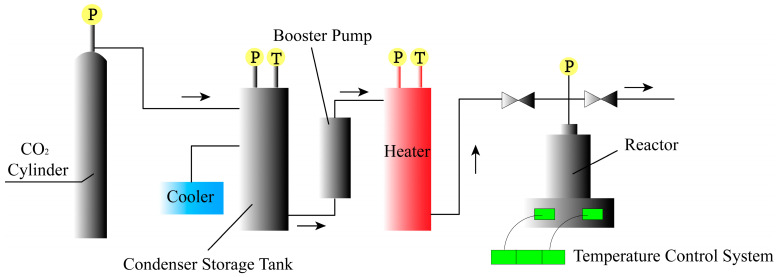
The process of preparing foam particles by the batch method (The letter P stands for adjustable pressure, T stands for adjustable temperature, and the arrows indicates the direction of flow of CO_2_).

A supercritical fluid reactor (HL-5L/25MPa) was purchased from the Hangzhou Huali Pump Industry Co., Ltd. (Hangzhou, China) (Figure 1). The electric heating (blast) constant-temperature drying oven (101A-1) was bought from Jiangsu Taizhou Tiantai Electric Heating Instrument Factory (Taizhou, China). The water-bath heater (SH-RA) was obtained from Shanghai Suhao Electric Co., Ltd. (Shanghai, China) for heating any foaming equipment containing water or glycerol.

### 2.3. Experimental Process

**Figure 2:**
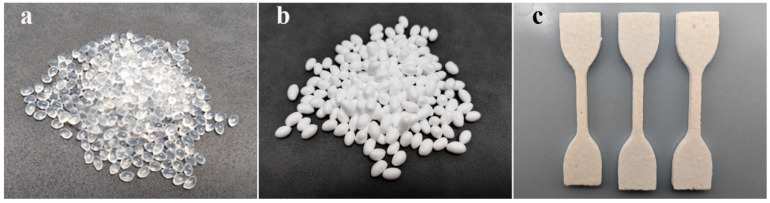
(**a**) Unfoamed ETPU beads. (**b**) ETPU foam beads after supercritical carbon dioxide foaming. (**c**) Sample strips pressed from foamed ETPU.

The TPU particles (Figure 2a) were first packed into a non-woven bundle bag, then the bag was tied shut and put into the reactor. With the lid closed, CO_2_ was blown into the reactor to remove the air inside. After confirming that the reactor did not leak, the reactor’s body was pressurized and heated; the saturation pressure was controlled to be 8 MPa, and the saturation temperature was controlled to be 37.5 °C [49]. Subsequently, the valves and instruments for the whole process were closed, and a constant temperature and pressure were maintained for 3 h so that the ScCO_2_ could completely permeate the TPU material. After 3 h had elapsed, the pressure was released at a pressure-releasing rate of 20 MPa/min until the gas in the reactor was completely drained, then the TPU particles were taken out and placed in the prepared foaming equipment for foaming.

Three different heating methods can be used for heating and foaming, namely: hot-air heating, water-bath heating, and glycerol-bath heating; the corresponding ETPU beads are named ETPU-air, ETPU-water, and ETPU-glycerol. When no more gas leaked out from the equipment, the foaming process ended. After cleaning the foaming media and drying was performed, ETPU beads (Figure 2b) were obtained. The ETPU beads were processed into a cuboid material (Figure 2c) via steam-chest molding. The molding parameters were controlled in the same way to avoid the influence of the processing factor on the ETPU’s mechanical properties: the mold cavity was first filled with the as-prepared ETPU beads; successively, water steam with a pressure of 3.2 bar and a temperature of 140 °C was introduced into the mold; after cooling to a lower temperature with cold water, the cuboid samples were obtained.

### 2.4. Testing and Characterization

The microscopic vesicle structure of the ETPU foamed beads produced under different foaming conditions was observed by scanning electron microscopy (SU8600, Hitachi Limited Co., Ltd. (Tokyo, Japan)). The ETPU foam beads with intact foaming quality were taken, and thin slices of cross-section (about 1–3 μm) were cut along the largest diameter of the foam bead grains with a razor blade and pasted on conductive adhesive, ready for observation.

In order to compare the mechanical properties of different ETPU materials, the ETPU beads were first processed into cuboid material (Figure 2c) via steam-chest molding. The densities of the different particle samples were measured by a specific gravity meter (DH-300, Dongguan Dahong Meituo Co., Ltd. (Dongguan, China)). The hardness was obtained using a Shore C durometer, according to GB/T 531.1-2008. Rebound rates were tested via drop ball methods, using a drop ball rebound tester (PK-262P, Dongguan Pinke Equipment Co. Ltd. (Dongguan, China)). The shrinkage rate was tested using a rubber compression permanent deformation machine, according to GB/T 6669-2008. The samples were prepared in a cuboid shape of 50 mm × 50 mm × 25 mm; then, they were compressed to 50% of their initial height (*d*_0_) with the machine and stored at 70 °C for 22 h; after cooling to room temperature for 30 min, the final height (*d*_1_)was measured and the shrinkage rate could be calculated using the following equation:

Shrinkage rate = (1 − *d*_1_/*d*_0_) × 100%.

## 3. Results and Discussion

The physical properties of the three foaming media are quite different (Table 1), so we guess that the foaming parameters of the ETPU particles foamed in these three media must also be different. According to our previous experimental results, in order to obtain fully foamed and regularly shaped ETPU, the appropriate foaming temperature ranges are different: 100–120 °C for hot air, due to its significantly low heat capacity, 65–85 °C for the glycerol bath, and 75–95 °C for the water bath. Thus, ETPU beads were prepared at 5-degree Celsius increments, from the lowest appropriate foaming temperature to the highest appropriate foaming temperature, in the three different media. 

**Figure 3:**
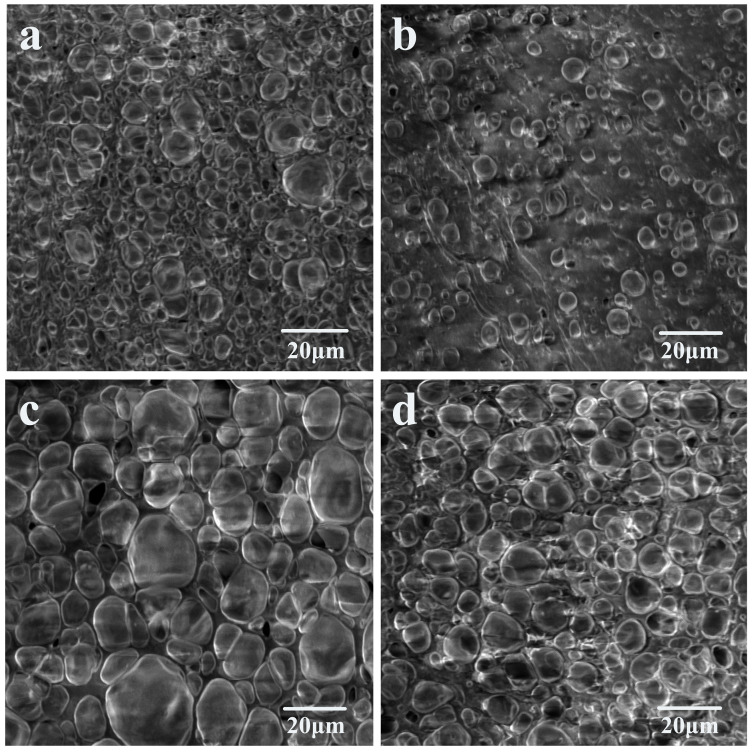
SEM images of ETPU beads after (**a**,**b**) hot-air foaming, (**c**) glycerol-bath foaming, and (**d**) water-bath foaming.

Scanning electron micrographs of the ETPU foamed beads produced by different foaming conditions are shown in Figure 3. The results showed that the bubble holes of the foamed bead particles prepared under hot air conditions were both large and small and were not uniform enough. The largest diameter of the bubble holes reached 15 μm (Figure 3a), while the smallest was only 2–3 μm (Figure 3b). This was caused by the uneven temperature distribution of the hot air in the oven. It was also observed at the same magnification that the bubble pore size of the ETPU foamed-bead grains obtained by foaming in glycerol was significantly larger than that of the ETPU foamed-bead grains obtained in a water bath. Most of the vesicles are between 10 and 20 μm in diameter and the size gap between the bead grains was also smaller than that of the bead grains obtained under hot air conditions (Figure 3c). This is mainly attributable to the heat capacity of glycerol being significantly greater than hot air, and the stability of the temperature in the liquid is also much higher than in air, which provides good foaming conditions for the foaming of TPU particles, and the foaming beads thus obtained are large and uniform. However, having a higher specific heat capacity of water than glycerol should have enabled the production of better ETPU beads than under the same conditions in glycerol, but because water can dissolve CO_2_, which plays a role in foaming, this decreases dramatically. As a result, the number of holes in the ETPU foamed beads that are foamed in water is lower than in the glycerol (Figure 3d). In order to further study the influence of foaming conditions on the performance of ETPU foam beads, this paper conducted mechanical property tests on ETPU foam beads obtained under different conditions.

### 3.1. Hot-Air Foaming

Hot air was acquired using a resistance wire to heat the air and was then used as the heating transfer medium. The thermal conductivity of air at 100 °C is 0.031 W m^−1^ K^−1^ and the specific heat capacity is 1030 J kg^−1^ °C^−1^. Here, it should be pointed out that the foaming temperature given for the hot air refers to the value set rather than the real-time temperature, as the evaporation process of ScCO_2_ is an endothermic process, which could lead to a decrease in the temperature. In addition, air convection when opening the oven can also contribute to the temperature decrease. Given the heat capacity and density of hot air, the real-time temperature might be 10 °C below the value set. As a matter of course, the oven temperatures for the water bath or glycerol bath also decrease, but the decrease is much smaller than with hot air. The foaming temperatures also refer to the value set.

**Figure 4:**
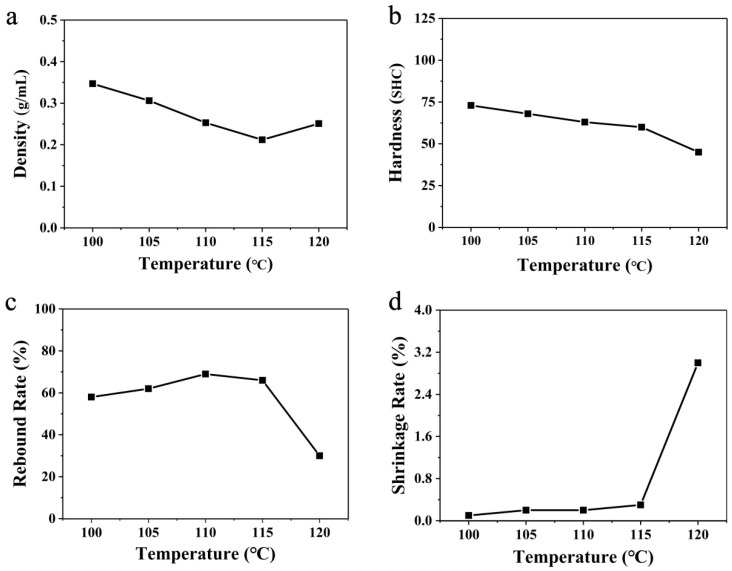
Linear variation of (**a**) density, (**b**) hardness, (**c**) rebound rate, and (**d**) shrinkage rate at different temperatures using hot-air foaming.

Typically, the foaming time for preparing ETPU-air is about 3 min. The surface of the ETPU beads prepared by hot-air foaming is shiny. However, the beads’ sizes are different because the temperature distribution of the hot air in the oven is very uneven for the endothermic evaporation of ScCO_2_ and the low heat capacity of hot air. The real foaming temperature deep inside the TPU beads would be much lower than the value set, which is not beneficial to producing large quantities of uniform ETPU goods. By reducing the amount of ScCO_2_-saturated TPU beads or by spreading the ScCO_2_-saturated TPU beads out sufficiently in the oven, the phenomenon of the ETPU beads’ uneven sizes could be improved (for example, the initial quantity of TPU particles is reduced to only half that in the other parallel experiments). To further investigate the properties of ETPU foamed in hot air, a series of experiments were carried out (Figure 4).

The density of the ETPU samples prepared in hot air decreased gradually at first with the increase in temperature and reached the lowest density of 0.212 g cm^−3^ at 115 °C. Nevertheless, the density increased again at 120 °C. When the ScCO_2_-saturated beads were placed in the hot air, heat transferred from the hot air to the beads, which would make the temperature of ScCO_2_-saturated beads rise. The test conditions were unable to satisfy the supercritical conditions of CO_2_. Thus, the unstable ScCO_2_ was either diffused through the TPU to escape into the environment or directly evaporated inside the TPU beads to form pores. It is obvious that the higher the temperature of the hot air, the more significant the evaporation process, which could make the pores larger and cause less CO_2_ to escape into the environment. Hence, as the temperature of the hot air increased to 115 °C, the density of the foamed beads gradually decreased. However, if the evaporation of ScCO_2_ is too severe, the pores would expand too markedly, which might break the pore walls, instead letting the CO_2_ gas escape. That is why the density of the foamed TPU increased again at 120 °C, and the mechanical properties would surely be affected by the broken pore walls. 

In order to investigate the relationship between foaming parameters, density, and mechanical performance, the hardness (Shore C), rebound rate, and shrinkage rate of the ETPU bulk prepared via steam-chest molding were tested. The hardness of the ETPU bulk samples gradually decreased as the temperature of the foaming media, hot air, increased to 115 °C. However, when the temperature reached 120 °C, the hardness of the corresponding sample further decreased noticeably to 45 SHC, although its density was higher than that of the sample prepared from the beads foamed in the 115 °C hot air. This hardness reflected the local resistance to deformation, which was related closely to the pore structure of ETPU foam in this case. As described above, the evaporation of the ScCO_2_ would become intense, which would inevitably make the pores larger and the pore walls thinner. Hence, the local resistance to deformation would decrease. Meanwhile, when the evaporation of ScCO_2_ was too severe in the 120 °C hot air, a considerable number of pores would burst to form a partial open-cell structure; the continuity of the pore walls was destroyed, and it was easy for the gas inside the ETPU to migrate or escape from the beads. As a consequence, although the density of the sample foamed in the 120 °C hot air was close to that of the sample foamed at 110 °C, its hardness was much less, which is not favorable for practical application. The rebound rate reflects the energy-storage capability of foam materials, which is determined by both the material’s mechanical properties and its pore structure (especially a closed-cell structure or open-cell structure): when the samples were compressed by the striking of the falling ball, the pores would deform and reduce the volume, and the kinetic energy of the falling ball would transfer to the potential energy of the bending pore walls and the compressed CO_2_ confined in the closed-cell pores, in addition to energy dissipation by the molecular friction of TPU and the escape of CO_2_. The rebound rates slightly grew as the temperature foaming media of the hot air increased from 100 °C to 110 °C; the rebound rate then slightly decreased at 115 °C, while still exceeding 66%; however, the rebound rate decreased dramatically to 30%, even less than half of that at 120 °C. These results also agree well with an explanation for the hardness: a considerable number of pores would burst to form a partial open-cell structure when the hot air reached 120 °C, due to the intense evaporation of unstable ScCO_2_. The shrinkage rate reflected the foam’s ability to resist permanent deformation. As can be seen in Figure 3d, the samples prepared with the foamed TPU beads in 100–115 °C hot air all possessed tiny shrinkage rates of less than 0.4%. However, the shrinkage rate of the sample prepared with the foamed TPU beads in 120 °C hot air apparently increased to 3.0%. The samples were all made of the same TPU, with the same creep deformation resistance; thus, their resistance to permanent deformation should be determined by the pore structure. With closed-cell pores, the gas cannot directly escape from the pore, and compressed gas with high pressure would help counteract the creep of the TPU pore walls. Meanwhile, open-cell pores cannot take advantage of compressed gas to inhibit the creep of TPU pore walls. That is why the shrinkage rate of the sample prepared with the foamed TPU beads in 120 °C hot air is much larger than those of the samples prepared at other temperatures, which are not suitable for long-life applications. In general, combined with the comprehensive properties of the material, it can be seen that the best foaming temperature for hot-air foaming would be between 110 and 115 °C.

### 3.2. Glycerol-Bath Foaming

For glycerol-bath foaming, glycerol was used as the foaming media. The thermal conductivity of glycerol is 0.45062 W m^−1^ K^−1^ and the specific heat capacity is 2100 J kg^−1^ °C^−1^; thus, glycerol stores much more heat than hot air, and the foaming temperature range (65–85 °C) for glycerol in the investigation would surely be lower than that in hot air. 

The first important appearance change brought about by the dramatic increase in the heat capacity is that the distribution of the foamed beads became more uniform. Due to the much higher heat capacity, the temperature distribution of the glycerol would not become markedly uneven during the endothermic evaporation of ScCO_2_. The real foaming temperatures for TPU beads at the outer or inner locations were nearly the same. Another change in appearance was that the surface of the foamed beads had a large number of uniformly distributed bubbles. Thanks to the much higher heat capacity, the medium temperature decreased, which could avoid severe evaporation of ScCO_2_ at the surface of the TPU beads and improve processability via the steam-chest molding process. These changes could benefit the industrial production of ETPU beads. However, glycerol is a kind of stick solvent with a high boiling point (290 °C, which is much higher than the viscous flow temperature of TPU), which makes it difficult to remove residual glycerol by simple filtration or evaporation; thus, it is essential to remove the glycerol with water before further processing. To further investigate the properties of ETPU that has been foamed in glycerol, a series of experiments were performed. 

**Figure 5:**
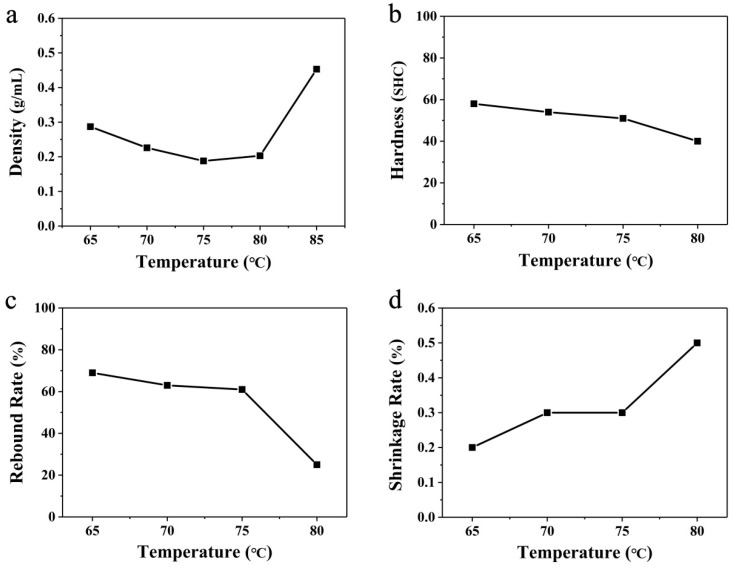
Linear variation of (**a**) density, (**b**) hardness, (**c**) rebound rate, and (**d**) shrinkage rate at different temperatures using glycerol-bath foaming.

From the broken line graph of the changes in the various parameters of the glycerol-bath foaming material at different temperatures in Figure 5, we can see that the density of the ETPU bulk samples prepared in the glycerol bath first decreased and then increased with the increase in the heating temperature of the glycerol bath, and then reached the minimum value (0.188 g cm^−3^) at 75 °C. The density of the material rose suddenly at a temperature of 85 °C. The trend of the densities of the ETPU bulk samples prepared in the glycerol bath in Figure 4a is similar to that in Figure 3a, implying that the density should also be determined by the pore size and the pore structure (either open-cell or closed-cell), which are affected by the heat transfer. As in the discussion above, the heat capacity of glycerol is much higher than that of air, which means that the ScCO_2_ could receive enough heat to evaporate at a much lower temperature. The minimum density of the ETPU bulk samples in glycerol was 0.188 g cm^−3^, which was slightly smaller than those produced in hot air. However, the temperature for the minimum density in glycerol was only 75 °C, which is much smaller than that of 115 °C in hot air.

Characterization of the ETPU bulk samples foamed in the glycerol bath was also carried out. It should be pointed out here that the ETPU beads foamed in the glycerol bath at 85 °C were stuck to each other (an ester change reaction might occur) so they were not able to be successfully processed into regular cubic samples. Therefore, the characterization was performed on the samples prepared at temperatures from 65 °C to 80 °C. The overall trend of the hardness (Shore C) of the ETPU bulk samples was also on a downward trend, as shown in Figure 4b, which is also similar to that in Figure 5a. Especially at a temperature of 80 °C, the hardness decreased obviously while the density started to increase, suggesting that the pore structures also started to change from a closed-cell to an open-cell structure. The rebound rate first decreased slightly with the temperature, and a significant decline also occurred in the rebound rate at 80 °C (Figure 5c), which agreed with the results for density and hardness. The shrinkage rate of the ETPU bulk samples showed an overall increasing trend in the temperature range of 65–80 °C (Figure 5d), among which the rise was relatively slow in the temperature range of 65–75 °C, but the segments rose sharply at a temperature of 80 °C, which is similar to the results given above for the ETPU samples prepared in hot air, which also implies that open-cell structures began to appear inside the ETPU beads prepared at 80 °C in s glycerol bath. In summary, it can be seen that this ETPU material has an optimized foaming temperature of 75 °C in a glycerol bath, which is much lower than 110–115 °C for hot-air foaming.

### 3.3. Water-Bath Foaming

The thermal conductivity of water is 0.62 W m^−1^ K^−1^ and the specific heat capacity is 4200 J kg^−1^ °C^−1^. Water, when used as the foaming media, was directly heated through a heating rod. Apparently, the heat stored by water is much greater than that stored by hot air; therefore, the foaming temperature range (75–95 °C) in the water bath to be investigated was surely lower than that for hot air. 

The ETPU beads were also relatively uniform, which is due to the much higher heat capacity of water. The real foaming temperatures for TPU beads at the outer or inner locations are nearly the same, just like in the glycerol bath, which could benefit the stability of the endothermic evaporation of ScCO_2_. Moreover, water is cheap, green, and easy to remove from the ETPU beads, and the resulting wastewater is also easy to recycle and purify, which are all positive attributes in industry. To further investigate the properties of ETPU when foamed in water, a series of experiments were also performed.

**Figure 6:**
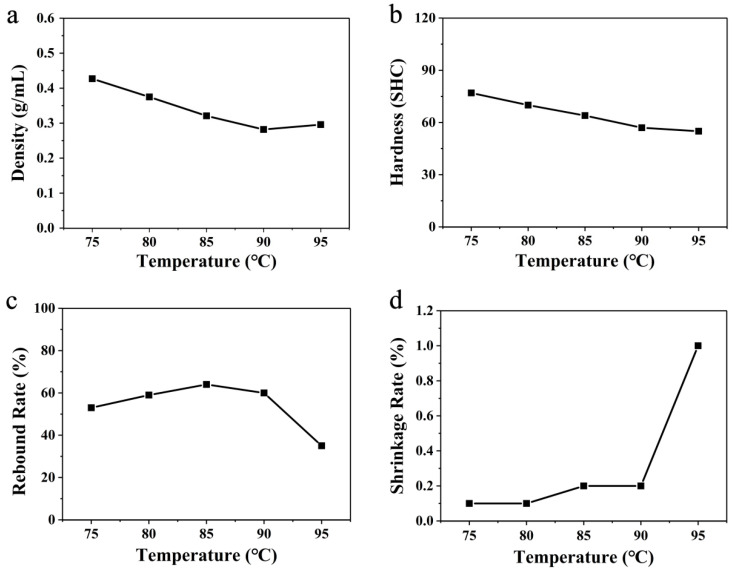
Linear variations in (**a**) density, (**b**) hardness, (**c**) rebound rate, and (**d**) shrinkage rate at different temperatures using water-bath foaming.

From the broken line graph with the changes in the various parameters of the water-bath foaming material at different temperatures, as shown in Figure 6, we can see that the density of the ETPU bulk samples prepared in the water bath decreased at first and then increased as the temperature increased, and reached the minimum value (0.282 g cm^−3^) when the temperature reached 90 °C. The rising and falling processes were relatively stable. The reason for this phenomenon could also be explained by the influence of the heating media on the evaporation of ScCO_2_ and the foaming process, as in the discussion about hot air and glycerol given above. It should be noticed that although the heat capacity of water is higher than that of glycerol, the minimum density of the ETPU beads foamed in water was much higher than that of the ETPU beads foamed in glycerol. This phenomenon might be caused by the dissolution of CO_2_ in water, which reduced the evaporation of CO_2_ in the form of gas, leading to an increase in the minimum density of ETPU beads foamed in water, compared to the other two foaming media. As a consequence, the mechanical properties of the corresponding ETPU bulk samples were also affected.

The hardness of the ETPU bulk samples has always been a decreasing trend with increasing temperature, but the decreasing trend tends to be slow during the period when the temperature is at 90–95 °C. As the foaming temperature increases, the rebound rate of the ETPU bulk samples increases at first and then decreases, and reaches the maximum when the temperature reaches 85 °C. This follows the same principle as for glycerol-bath foaming. The shrinkage rate of the ETPU bulk samples slowly increased from 75 °C to 90 °C, but suddenly rose at 95 °C. This result is consistent with hot-air foaming. In general, the best foaming temperature with water-bath foaming is 90 °C.

### 3.4. Comparison

**Figure 7:**
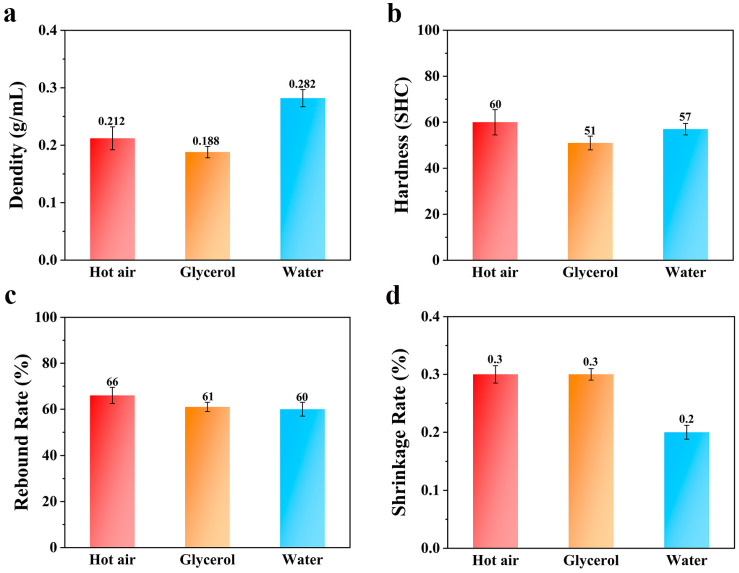
Columnar comparison of the (**a**) density, (**b**) hardness, (**c**) rebound rate, and (**d**) shrinkage rate of different media at the optimum foaming temperature.

The best foaming temperatures for hot-air foaming, glycerol-bath foaming, and water-bath foaming are 115 °C, 75 °C, and 90 °C, respectively. Figure 7 shows the results obtained by comparing the performance differences at the optimal foaming temperature of each medium.

According to Figure 7a, at the optimal foaming temperatures of each medium, the density of the foam material obtained by water-bath foaming was the largest, followed by hot-air foaming, and the density of the foam material obtained by glycerol-bath foaming was the smallest. This is due to the larger specific heat capacity of glycerol; the thermal conductivity of its TPU particles in the process of foaming can provide more heat, and there is a greater specific heat capacity of water due to dissolved CO_2_, but this can lead to conditions in the foaming of ETPU beads giving greater density. Because the TPU material has a certain water absorption capacity, in the process of water-bath foaming, TPU is likely to swell due to water absorption, which reduces the strength of the material surface. In the process of carbon dioxide release, the cells break, and the density increases. According to Figure 7b, the hardness of the foam material obtained by hot-air foaming and water-bath foaming was equivalent, and the hardness of the foam material obtained by glycerol-bath foaming was the smallest, which is due to its relatively low density. Figure 7c shows a comparison of the rebound rate of each medium at the optimal foaming temperature. The rebound rate of the foaming materials obtained by glycerol-bath foaming and water-bath foaming was comparable, while the rebound rate of the foaming material obtained by hot-air foaming was the largest. This is due to the fact that the foaming resistance of the TPU material in hot air was smaller than that of samples produced in glycerol and water. Figure 7d shows that the shrinkage rates of the three media at the optimum foaming temperature were almost the same, which indicates that as long as the optimum foaming temperature of the medium is maintained, the choice of medium has little effect on the shrinkage rate of the TPU foaming material.

## 4. Conclusions

In this paper, the foaming time of water and glycerol-bath foaming was reduced to 2/3 of the hot-air foaming time to improve the foaming rate of TPU greatly. The best foaming temperatures of hot-air foaming, glycerol foaming, and water foaming are 110–115 °C, 75 °C, and 90 °C, respectively. There was no introduction of new impurities in hot-air foaming compared to glycerol- and water-bath foaming. The foaming density of glycerol-bath foaming was the smallest, followed by hot-air foaming, and the foaming density of water-bath foaming was the largest. The material hardness of glycerol-bath foaming, water-bath foaming, and hot-air foaming increased successively, and the hardness difference between water-bath foaming and hot-air foaming was small. The resilience of glycerol-bath foaming and water-bath foaming materials was basically the same, while the resilience of glycerol-bath foaming material was greater than those made using these two methods. After the glycerol-bath foaming and hot-air foaming particles were static for the same period of time, the shrinkage rate was the same, and the shrinkage rate of water-bath foaming was less than with these two methods. Finally, by comparing the performance of the three media at the best foaming temperature, it is evident that no matter what kind of medium is used, as long as the best foaming temperature is found, the performance of the foamed material that is obtained is not much different. Given production stability and economy, a water bath should be an acceptable choice in most situations. Otherwise, for some special requirements, we would recommend using the glycerol bath for a less dense foam. The above results provide a reference for choosing the appropriate foaming method according to the actual requirements. 

## Figures and Tables

**Table 1:** Physical properties of the three types of foaming media (hot air, glycerol, and water).

Foaming Media	Hot Air (100 °C)	Glycerol	Water
Thermal conductivity (W m^−1^ K^−1^)	0.031	0.45062	0.62
Specific heat capacity (J Kg^−1^ °C^−1^)	1030	2100	4200

## Data Availability

The original contributions presented in the study are included in the article, further inquiries can be directed to the corresponding author.
